# The Effect of the Addition of Sage (*Salvia officinalis*) and Lucerne (*Medicago sativa*) on the Strength Parameters of a Polymer-Based Composite and Socio-Economic Analysis

**DOI:** 10.3390/ma18132959

**Published:** 2025-06-23

**Authors:** Nikolina Poranek, Marcin Marczak, Agata Wajda, Krzysztof Pikoń

**Affiliations:** 1 Department of Technologies and Installations for Waste Management, Faculty of Energy and Environmental Engineering, Silesian University of Technology, Konarskiego 18, 44-100 Gliwice, Poland; krzysztof.pikon@polsl.pl; 2 Faculty of Mechanical Engineering, Silesian University of Technology, 44-100 Gliwice, Poland; marcmar690@polsl.pl; 3 Unirubber Sp. z o.o., Zielonka 17, 59-940 Węgliniec, Poland; 4 Institute of Energy and Fuel Processing Technology, 41-803 Zabrze, Poland; awajda@itpe.pl

**Keywords:** polymer-based composite, durability, strength parameters, plant filler, lucerne, rubber, sage

## Abstract

Polymer composites are of considerable interest due to the possibility of improving the performance parameters of plastics. The filler is a component whose introduction into the rubber mixture can affect the physicochemical and functional properties of the composite. It is present in the largest quantity in the mixture, so its type is of significant importance in the polymer composite production process. Currently, much attention is being paid to the potential use of various materials as fillers to improve the properties of composites. These materials should, among other things, exhibit good adhesion to the polymer matrix and a high degree of dispersion. One example of such a material is dried plant material. In this group, dried leaves of two plants—sage (*Salvia officinalis*) and lucerne (*Medicago sativa*)—were introduced into a rubber mixture in several different content variants. The mixtures were subjected to durability and aging tests and the results were compared with a mixture without any plant additives. Of all the test variants with plant filler, the best results were obtained with the lowest proportion of dried plant material, which was 5 Parts per Hundred Rubber (PHR). In this case, most parameters remained at a level similar to the variant without additives. A slight improvement was observed in elongation at break for the mixture with sage (from 550% to 559%), while in the case of the mixture with lucerne, the color improved (from 1.21 to 0.94). Some parameters of vulcanization characteristics and tensile strength deteriorated. For the latter parameter, a decrease of 11% was noted for the mixture with sage (from 4.65 MPa to 4.13 MPa) and 18% for the mixture with lucerne (to 3.82 MPa). Interestingly, as a result of the ageing of the samples, a significant part of the mixtures with dried plants obtained better results in the case of tensile strength than before ageing. This applies especially to the following variants: 30 PHR for the mixture with sage (an increase of 48%) and 5 PHR for the mixture with alfalfa (an increase of 15%). In general, it should be noted that the functional parameters deteriorated with the increase in the proportion of plant additives.

## 1. Introduction

Polymer composites are of particular interest to industry. This is due to their specific properties obtained by combining at least two materials with differing characteristics [1,2,3]. Among the advantages of polymer composites, compared to monolithic materials, the most commonly cited are improved corrosion resistance, increased stiffness, and reduced weight [4,5]. A significant component of the mixture, making up the largest portion, is the filler, whose introduction into the formulation affects the physicochemical, processing, and functional properties of the resulting composites [6,7]. Currently, great importance is placed on the introduction of so-called bio-fillers (renewable or recycled materials) into the mixture, which can fully or partially replace industrially used fillers. This trend has become particularly visible in recent years, marked by an increase in environmental awareness [8,9,10]. It allows for a reduction in the use of plastics or rubber in composite production. The substitution of fossil fuels or non-renewable materials with renewable materials fits into the idea of a circular economy, as well as the sustainable development goals and climate change mitigation objectives set by the United Nations [1,2].

In the production of biocomposites, the following raw materials are most commonly used: palm oil waste, flax and hemp fibers, rice husks, kenaf fibers, coconut shells and fibers, as well as dry mass from sugarcane [9,11]. Moreover, increasing attention is being paid to commonly occurring and non-demanding herbaceous plants [12]. Natural origin fillers have many advantages that are important from the perspective of composite production. They are widely available, their acquisition cost is low, and they are suitable for recycling. Furthermore, the properties of these materials positively affect the functional parameters of the product [13,14,15]. Composites containing natural origin materials are environmentally friendly and find applications in various industries, including the construction, automotive, transportation, sports, and packaging production industries [16].

The development of the biocomposites sector is best reflected by the situation of the global market, with their production and sale having steadily increased. The steadily expanding field of their applications is also noticeable in the forecast. In 2021, the global biocomposites market was worth USD 24.59 billion. It is expected to grow to USD 87.73 billion by 2030, at a Compound Annual Growth Rate (CAGR) of 16.8 per cent over the forecast period 2022–2030. A large contributor to this is the automotive industry, where the automotive composites market will reach almost USD 16 billion by the end of 2030 [17].

Fillers should show good adhesion to the polymer matrix and should have a high degree of dispersion. They should not contain crystalline or surface water [18,19]. The above-described requirements are met, among others, by dried sage (*Salvia officinalis*) and lucerne (*Medicago sativa*), which are the subject of the analyses presented in this article. These are widespread herbaceous plants that do not require special cultivation conditions and are therefore characterized by their high availability and low cost. The effect of the addition of sage to the composite was studied by [20,21,22,23], among others. Słota et al. [23] developed a ceramic–polymer composite with sage extract for the regeneration of bone tissue. In [21], the properties of rigid polyurethane foams using sage filler were analyzed. In the first case, satisfactory results were obtained with regard to biocompatibility, biodegradability, and the possibility of the gradual release of active ingredients over time. In the case of polyurethane foam, improvements in performance were recorded at lower proportions of sage fillers.

The aim of this work is to investigate the effects of sage and lucerne fillers on an ethylene propylene diene monomer (EPDM) polymer-based composite. Different proportions of fillers were used in the mixtures, which were subjected to strength tests. Recent advancements have been observed in the development of polymer composites incorporating both biodegradable and non-biodegradable alternatives to conventional fossil-based polymers [24]. However, the exploration of plant-derived additives for application in polymer-based composite systems remains relatively limited. There is a recognized need for expanded research to evaluate the compatibility and functional properties of polymer mixtures formulated with plant-based fillers [25].

The plant-derived additives investigated in this study have previously been examined in other research contexts. Członka et al. [24] utilized sage extract modified with montmorillonite as a bio-additive, demonstrating its potential to reduce the flammability of rigid polyurethane foams. Miedzianowska et al. [25] assessed the effects of incorporating lucerne into rubber. The plant additive was introduced in multiple forms, including biomass, phyto-ashes, and plant extracts. For the biomass variant, three proportions were employed: 10 PHR, 20 PHR, and 30 PHR. The authors of this study decided to use both types of herbs as bio-additives. Experimental investigations were conducted on polymer-based mixtures containing four different proportions of sage and lucerne: 5 PHR, 10 PHR, 20 PHR, and 30 PHR.

## 2. Materials and Methods

### 2.1. Materials

Laboratory tests were carried out using a laboratory mixer for mixtures based on EPDM, ethylene norbornene (ENB), and low-density polyethylene (LDPE). Calcium carbonate with a density of 2.7 g/cm^3^ and a low moisture content of <1.0% was used as a filler. The plastic-fixing agent was paraffin oil with a density of 0.87 g/cm^3^ and a kinematic viscosity of 108.1 mm^2^/s (at 40 °C). The material had a paraffin content of 69%, a naphtha content of 31%, and a sulfur content of 0.002%. The vulcanization activator was zinc oxide, which is used during typical sulfur vulcanization. Vulcanization accelerators are typical for sulfur-curing vulcanization systems. The pigment was iron oxide with RAL 3016, pH = 4–8, and a density of 5.25 g/cm^3^. The crosslinking agent was sulfur; it was non-oil-based, rhombohedral, light yellow in color, and had a pH of 6.6. The melting point of the agent ranged from 111 to 119 °C and the bulk density was 550–750 kg/m^3^.

Table 1 presents the components of the control sample, which is rubber without the addition of dried plant materials.

**Figure 1:**
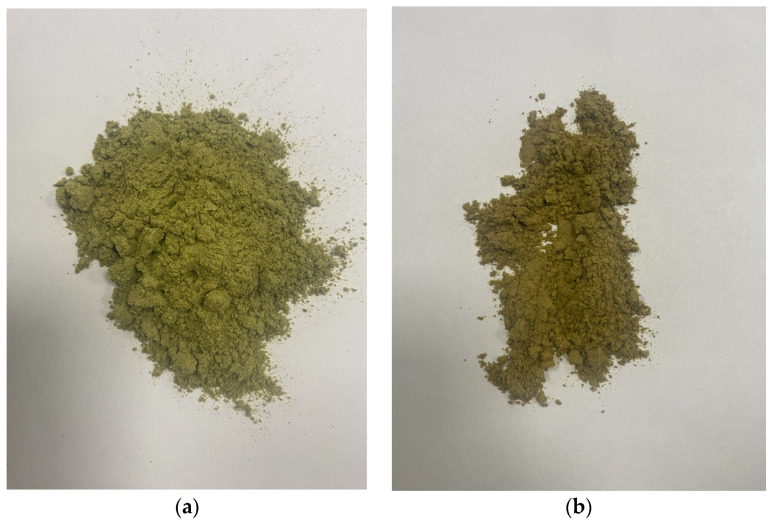
Dried organic materials used in the mixture: (**a**) sage (*Salvia officinalis*); (**b**) lucerne (*Medicago sativa*).

Dried, above-ground parts of sage (*Salvia officinalis*) and lucerne (*Medicago sativa*) were selected for analysis. The air-dried plants were then ground in a ball mill. Figure 1 shows the organic materials used in the analysis.

Determining proportions of the tested plant substitutes in the mixture depends on many factors. In studies of this type [24,25,26,27], the proportions of biomass additives of 5 PHR, 10 PHR, 15 PHR, 20 PHR, and 30 PHR are often found. In this work, a fairly wide range of plant additive mixture shares was also established, defined here as 5 PHR, 10 PHR, 20 PHR, and 30 PHR. Table 2 summarizes the proportions of the individual components of the mixtures with the dried plant materials tested in the following variants: 5 PHR, 10 PHR, 15 PHR, and 20 PHR.

### 2.2. Preparation of Samples for Testing

The rubber mixtures were prepared under laboratory conditions in a Brabender PlastiCorder tangential rotor mixer (Brabender GmbH & Co. KG, Duisburg, Germany) in a one-ethereal mixing cycle. The mixing process was controlled continuously according to a defined mixing regime:

1. Dosing of polymer (EPDM), filler (calcium carbonate), and plasticizer (paraffin oil), along with dried plants, into the mixer chamber.
2. Lowering the vertical wedge and setting the rotor speed of the mixer to 25 revolutions per minute (RPM).
3. Continuous mixing for 60 s (achieving a mixing temperature of approximately 70 °C).
4. Raising the vertical wedge, manually cleaning the pressure stamp of the mixer, and dosing the previously prepared crosslinking agent at approximately 70 °C.
5. Increasing the rotor speed to 30 RPM and mixing for 60 s.
6. Manually cleaning the mixer chamber, increasing the rotor speed to 35 RPM, and mixing for 60 s.
7. Mixing the rubber mixture (40 RPM) until the temperature reaches 95 °C.
8. Stopping the laboratory mixer motor, opening the chamber, and removing the prepared rubber mixture after approximately 5.5 min of mixing.

The prepared rubber mixtures were slabbed for 4 min to a thickness of 6 mm on a Polymix 110 L laboratory roller mill (Servitec, Wustermark, Germany). The temperature of the rollers was 50 °C (controlled by a temperature control unit (TCU) system to maintain the imposed roller temperatures) and the rollers were rotated at a preset friction of 1:1.2, at a speed of 20 RPM. After flotation, each rubber mixture was labelled for quick identification. The sample was labelled with the date of mixing and the mixture variant. The mixture was left to condition for 24 h in a room where the relative humidity was kept constant at (35 ± 5)%. Samples were taken for physico-mechanical testing of the prepared material.

**Figure 2:**
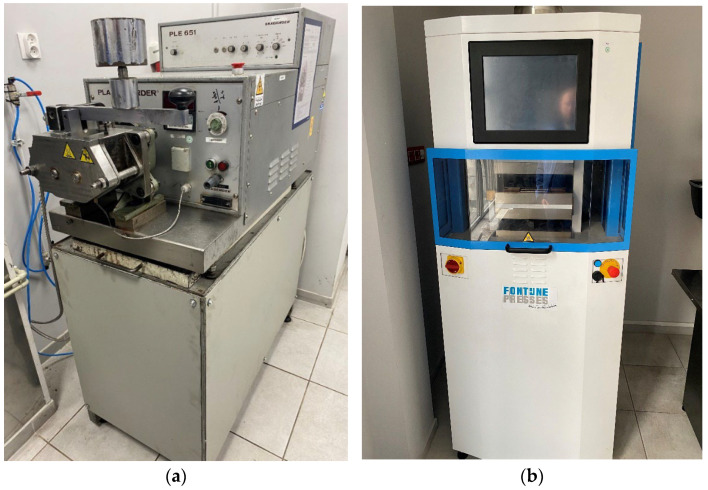
The equipment used in the preparation of the samples: (**a**) Brabender PlastiCorder mixer; (**b**) Fontijne laboratory press.

The rubber mixture samples were then subjected to thermal vulcanization in a Fontijne laboratory press. The samples were vulcanized under a pressure of 10 MPa for 30 min at a temperature of 160 °C in special vulcanization molds. After vulcanization, the samples were cut using a cutting press equipped with a die, compliant with the PN-ISO 37-1 standard, into the shape of so-called paddles. The equipment used in the preparation of the samples for the strength tests is shown in Figure 2.

**Figure 3:**
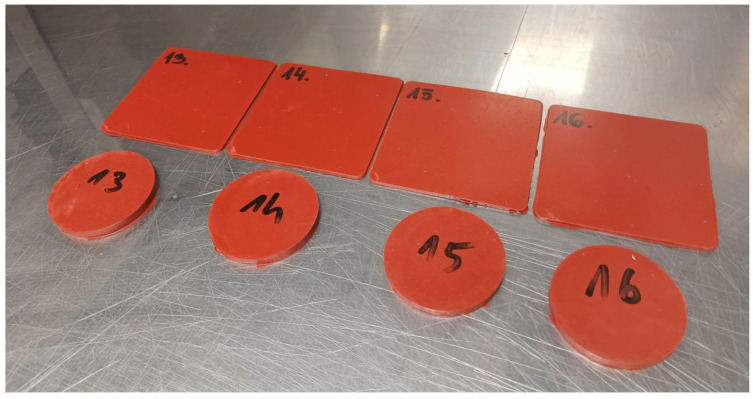
The view of the samples prepared for strength test.

The preparatory steps resulted in the formation of samples, as shown in Figure 3.

### 2.3. Methods

The properties of the materials tested were determined in the following ranges and according to the following procedures:

- Mechanical strength.

The mechanical properties of the produced composites were tested using a ComeTech testing machine (ComeTech, Taichung City, Taiwan) (hardness tester), in accordance with the PN-ISO 37 standard, using standard paddle-shaped samples. The samples were subjected to a stress–strain test at a crosshead speed of 500 mm/min, with measurements taken at room temperature. Based on the obtained stress–strain curve, the tensile strength (TSb) and elongation at break (Eb) were determined. The results from 5 samples for each composite were averaged.

- Specific weight.

The determination of the specific weight was carried out using a PS360.R2 hydrostatic scale (densymeter) (RADWAG, Radom, Poland) by immersing a sample of unvulcanized rubber compound in distilled water, in accordance with PN-ISO 2781:1996.

**Figure 4:**
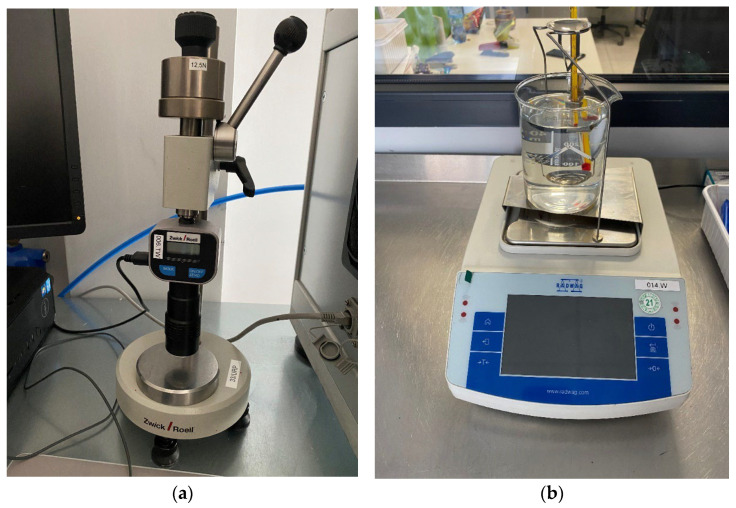
Laboratory stands: (**a**) hardness tester; (**b**) hydrostatic scale.

Figure 4 shows the equipment used to test material properties: hardness (Figure 4a) and specific gravity (Figure 4b).

- Vulcanization characteristics.

The vulcanization characteristics of the rubber mixture were studied using a Moving Die Rheometer (MDR) from TA Instruments (New Castle, DE, USA). After cutting the samples on a press and calculating the mass to fill the vulcameter chamber, the samples were subjected to vulcanization at 175 °C for 10 min, in accordance with PN-ISO 6502. From the registered vulcanization curve, which can be defined as the relationship between torque and time, *M* = *f*(*t*), the following torque values are determined:

ML—Minimum torque value [dNm];
MH—Maximum torque value after a specified time [dNm].

Time values:

t50—The vulcanization time at which the torque reaches 50% of the total torque (50% vulcanization degree), according to the following formula:
  *t*50 = *ML* + 0.5*∙*(*MH* − *ML*)

ts2—The time after which the torque increases by 0.2 Nm above the ML value [min];
t90—Vulcanization time, at which the torque reaches 90% of the total moment:
  *t*90 = *ML* + 0.9*∙*(*MH* − *ML*)

- Kinematic viscosity.

The kinematic viscosity of the rubber compounds was measured using a shear disk viscometer, model MV one (TA Instruments). The method involves measuring the torque required to maintain the rotation of a metal rotor in a cylindrical chamber. This chamber consists of two dies filled with a rubber compound. The resistance exerted by the mixture is expressed in conventional units—Mooney degrees—which characterize the viscosity of the mixture being tested. The samples, after being cut out using a cutting press and recalculated by weight to fill the viscosity-meter chamber, were subjected to the test. For the rubber compound viscosity test, defined as ML(1 + 4)100, the individual symbols stand for the following:

M—Method developer, Mooney.
L—Large rotor; it is also possible to use the S (small) rotor for materials with low specific density.
1—Preheating time before starting the rotor, [min].
4—Test duration counted from rotor start to final reading, [min].
100—Test temperature, [°C].

**Figure 5:**
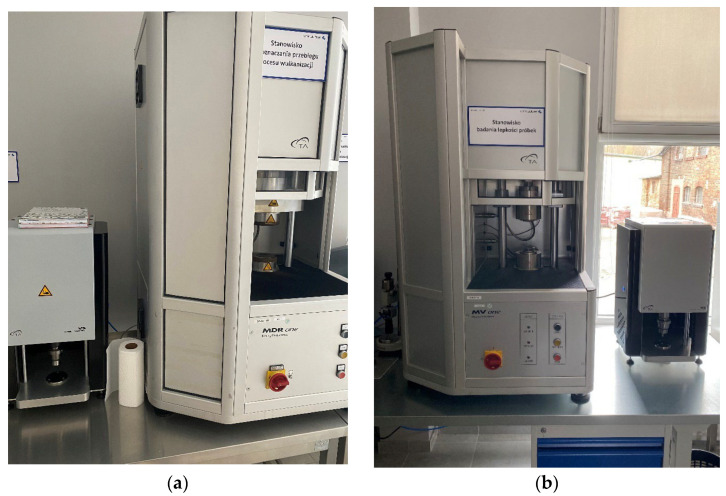
Laboratory stands: (**a**) for vulcanization test; (**b**) for viscosity test.

Figure 5 shows the equipment used to test the material properties: vulcanization (Figure 5a) and viscosity (Figure 5b).

- Colorimetric properties.

Testing of the colorimetric properties of the produced vulcanized composites was carried out with a Chroma Meter CR-410 colorimeter using SpectraMagic NX software (ver. 3.40). The results of the color tests were compared to a standard (reference, red-RAL 3016, rubber compound sample).

- Aging of the samples.

The samples for strength and hardness tests were subjected to elevated temperature air in an air-circulating oven (method B). The aging conditions are defined by ISO 188, while the strength test follows ISO 37, and the hardness test is conducted according to ISO 38. The aging of paddle-shaped samples was carried out for 70 h at a temperature of 135 °C.

- Conditions of physicochemical tests.

The physico-mechanical tests of all the composites were conducted under temperature (21–25 °C) and humidity (40–60%) conditions controlled by a calibrated thermohygrometer.

## 3. Results

**Figure 6:**
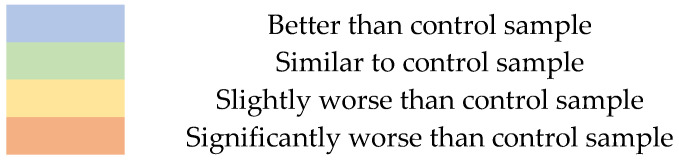
Evaluation of the properties of the tested samples.

The results of the strength tests of the rubber samples with the addition of plant dry matter—from sage and lucerne—are presented in Table 3. A classification of their properties is also introduced, based on the percentage change in the parameter values compared to the control sample, as well as the quality of the change. The color coding was adopted according to the principle shown in Figure 6.

The better-than-control-sample assessment is used for a positive change in the value of a given parameter. This change, depending on the character of a given parameter, can take a negative or positive value. The range of the change value was not specified here—any percentage change in a given parameter to a more favorable one is considered a change for the better. The remaining three assessments—similar, slightly worse, and significantly worse—are only applicable to cases presenting worse results than samples without additives. In addition, a range of percentage change value is specified for each of these assessments. Similar-to-control-sample assessment is provided when the result of a given parameter is less favorable and the percentage change in value is within the range of up to +/−5%. Similarly, the slightly worse assessment covers a percentage change in value greater than +/−5% to +/−10%, while the significantly worse assessment is used for results characterized by a percentage change in value greater than +/−10%.

Among the variants of dried plant content in the samples, the best strength parameters are observed with the lowest one, namely 5 PHR. This applies to both sage and lucerne dried materials. Compared to the control sample, a deterioration in the values of almost all parameters is observed. However, it can be concluded that the addition of dried plant matter at the smallest proportion (5 PHR) does not significantly worsen the performance parameters of the rubber, while improving some of them. The properties that are improved by the plant additive include elongation at break (Eb) (for samples with sage added at 5 PHR and 10 PHR, and also for samples with lucerne added at 10 PHR) and color (for samples with sage added at 10 PHR, and also for samples with lucerne added at 5 PHR).

**Figure 7:**
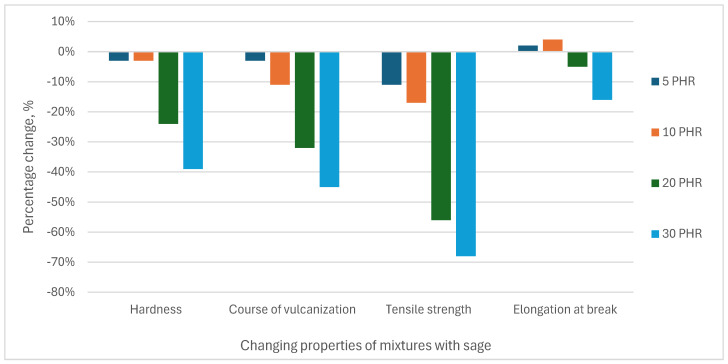
Percentage changes in properties of mixtures with plant additives in relation to the mixture without additives—results for sage.

**Figure 8:**
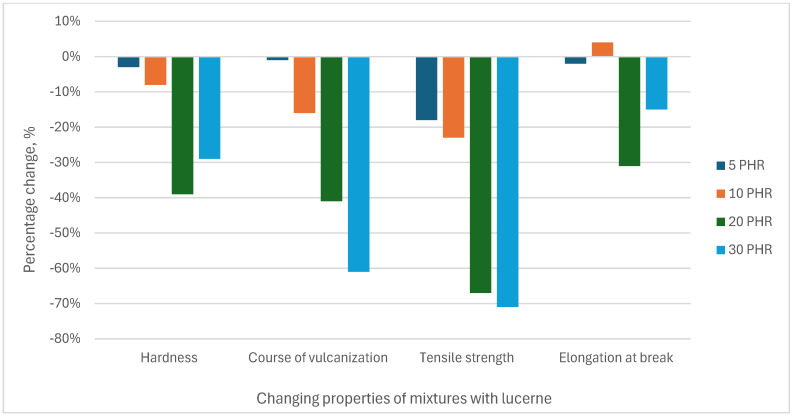
Percentage changes in properties of mixtures with plant additives in relation to the mixture without additives—results for lucerne.

The percentage changes in selected properties of the mixtures with plant additives in relation to the mixture without plant additives are presented in Figure 7 (sage) and Figure 8 (lucerne).

**Figure 9:**
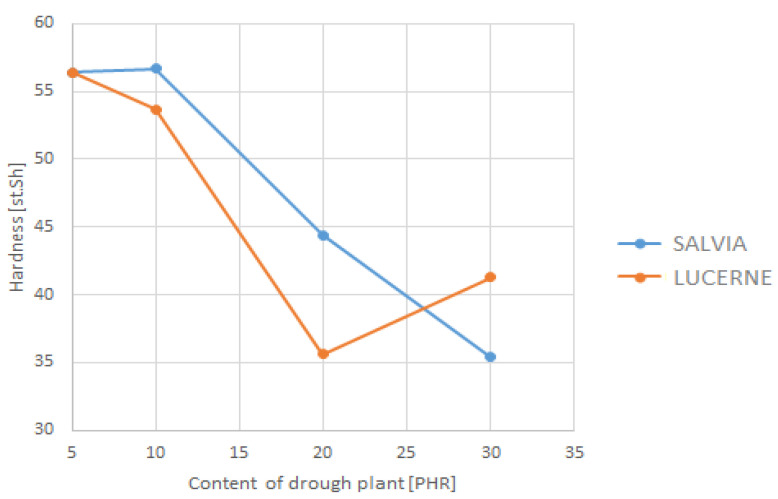
Dependence of hardness on dried plant content.

**Figure 10:**
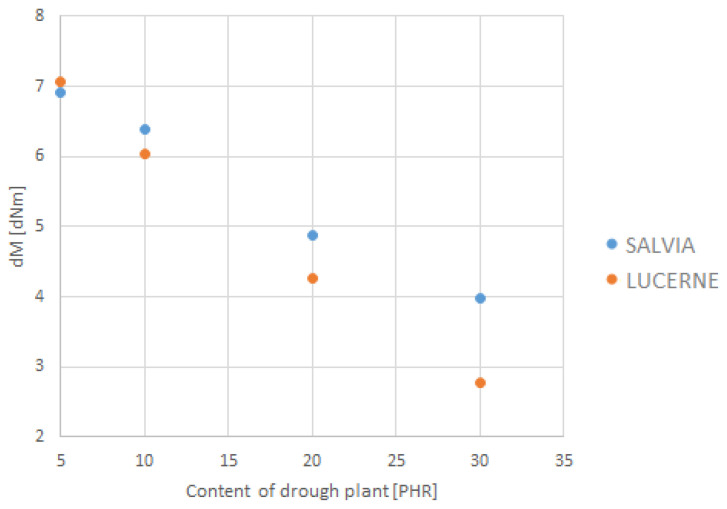
Dependence of the course of vulcanization on the content of dried plant matter.

**Figure 11:**
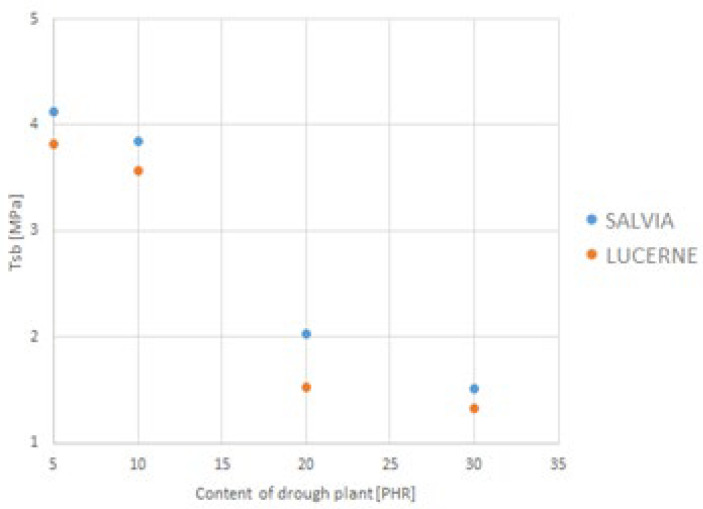
Dependence of tensile strength on dried plant content.

**Figure 12:**
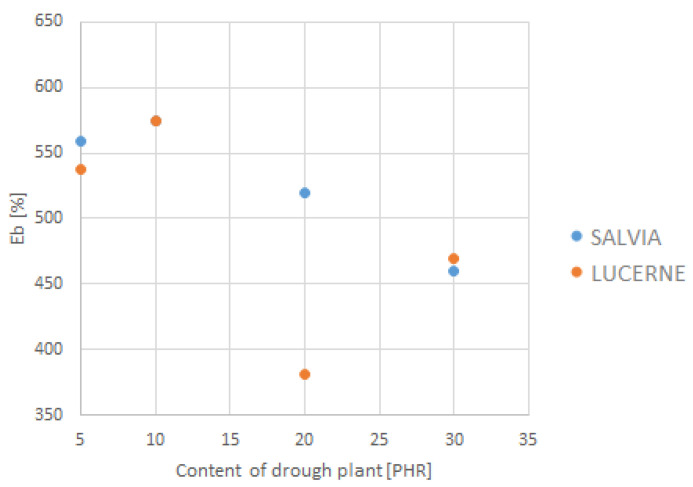
Dependence of elongation at break on dried plant content.

The depiction of the relationship between the selected strength parameters and the content of dried plant matter is shown in Figure 9, Figure 10, Figure 11 and Figure 12. The data for the graphs are provided in Table 4.

The properties of the tested samples were also examined after simulated aging. The results of the aging test are presented in Table 5.

The results after ageing are somewhat surprising. The control sample showed a deterioration in values for two parameters—hardness and elongation at break. Tensile strength remains almost unchanged, while color improves significantly. In the case of the dried plant samples, most of the parameters worsened, but a significant increase is observed for most of the dried plant matter proportions in the case of tensile strength. This is the parameter for which no improvement was observed in the properties of the samples before the simulated ageing process. The probable mechanism responsible for this improvement is the internal migration of components within the material system. Ageing may promote the intensification of the adhesion of the lignocellulosic phase to the polymer matrix, which leads to the increased mechanical integrity of the composite.

## 4. Socio-Economic Analysis

The incorporation of plant-based fillers, such as sage (*Salvia officinalis*) and lucerne (*Medicago sativa*), into rubber composites not only influences material properties, but also has far-reaching socio-economic implications. This shift towards bio-based materials aligns with global sustainability goals and offers both direct and indirect benefits to various stakeholders, from farmers to manufacturers and consumers.

The integration of plant-based fillers, such as *Salvia officinalis* and *Medicago sativa*, into rubber composites represents a strategic convergence of materials science, environmental stewardship, and socio-economic development. Beyond the immediate environmental benefits—including reduced reliance on non-renewable resources and lower carbon emissions—this transition holds the potential to stimulate local economies, foster job creation, and fortify supply chain resilience. By replacing synthetic fillers with renewable alternatives, manufacturers can mitigate market volatility, align with evolving regulatory landscapes, and future-proof their operations against the rising costs of fossil-based materials.

Moreover, the adoption of bio-fillers opens avenues for technological innovation and market differentiation. While current trade-offs in mechanical performance necessitate further research and optimization, continuous advancements in material processing and hybrid filler systems could bridge this gap, enabling the production of high-performance, low-impact composites. As consumer demand and legislative pressure increasingly favor sustainable products, early adopters of bio-composite technologies will likely gain a competitive edge, accessing new revenue streams and strengthening brand value.

Ultimately, the shift towards bio-based fillers is not merely an environmental imperative, but a catalyst for systemic value creation. Through strategic collaboration between academia, industry, and policymakers, the widespread adoption of plant-derived fillers could drive a paradigm shift towards a truly circular economy—where material innovation is intrinsically linked to societal well-being and long-term economic resilience [28,29,30,31,32,33,34]. Table 6 shows the socio-economic benefits of sage and lucerne usage in polymer-based composites.

## 5. Conclusions

Based on the results obtained, it can be observed that dried plant matter can be considered as an alternative filler for highly filled EPDM compounds. The study examined the effect of using dried sage and lucerne, which, depending on their proportion in the mixture, modify the physico-chemical properties of the composite. The degree to which the properties of the rubber mixture change depends on the amount and type of dried plant matter used. The results showed that the best performance parameters were obtained for the smallest proportion of dried plant matter, namely 5 PHR, for both sage and lucerne. Improvements in some of the performance parameters were also observed for a lucerne content of 10 PHR. The parameters that improve with the addition of the tested plants are color and elongation at break, while most of the performance parameters do not change significantly for mixtures with a 5 PHR plant content. Nevertheless, it should be noted that, in general, mixtures containing dried plant matter present lower quality in most performance parameters. Based on these studies, the following trend can be observed: the higher the share of dried plant matter in the mixture, the worse the performance parameters. On the other hand, it should be noted that a small share of dried plant matter does not significantly affect the deterioration of performance parameters, and in some cases improves them. Hence, a general conclusion can be drawn indicating good prospects related to the inclusion of a small amount of dried plant matter in the mixture. Interestingly, the aging simulation carried out for half of the test variants showed a significant improvement in one parameter in relation to the control sample after aging, namely tensile strength. The probable cause of this phenomenon is the migration of components within the formulation. Ageing can cause dried plant matter to adhere to the polymer matrix. This is a relevant subject that may provide a foundation for a comprehensive assessment of additive migration and interfacial compatibility within the polymer matrix. Socio-economic analysis has shown a number of advantages associated with the use of dried sage and lucerne as substitutes in these mixtures. The wide-scale use of this solution will have a positive impact on the environment, the development of local agriculture, and the diversification of supplies of raw materials necessary for production.

## Figures and Tables

**Table 1:** The content of individual components in the control sample.

Control Sample				
Component	SP. GR.	Mass [g]	Content [%]	PHR *
EPDM	1.23	31.90	7.09	41.70
ENB	1.23	44.60	9.91	58.30
Paraffin oil	0.87	63.00	14.00	82.35
Calcium carbonate	2.70	280.22	62.27	366.29
LDPE	2.30	6.75	1.50	8.82
Ferrum oxide	5.25	18.00	4.00	23.50
Stearic acid	1.80	1.17	0.26	1.50
Zinc oxide	1.80	0.98	0.22	1.30
Accelerators	0.91	2.11	0.47	2.70
Sulfur	1.22	1.29	0.29	1.70

* PHR (Parts per Hundred Rubber)—the weight of additives per 100 units of rubber.

**Table 2:** Composition of samples with the addition of dried sage and dried lucerne.

Samples with the Addition of Sage												
	5 PHR of Sage			10 PHR of Sage			20 PHR of Sage			30 PHR of Sage		
Component	M [g]	%	PHR	M [g]	%	PHR	M [g]	%	PHR	M [g]	%	PHR
EPDM	31.90	7.09	41.70	31.90	7.09	41.70	31.90	7.09	41.70	31.90	7.09	41.70
EPDM off spec.	44.60	9.91	58.30	44.60	9.91	58.30	44.60	9.91	58.30	44.60	9.91	58.30
Paraffin oil	63.00	14.00	82.35	63.00	14.00	82.35	63.00	14.00	82.35	63.00	14.00	82.35
Calcium carbonate	276.39	61.42	361.29	272.57	60.57	356.29	264.92	58.87	346.29	257.27	57.17	336.29
Poliethylene	6.75	1.50	8.82	6.75	1.50	8.82	6.75	1.50	8.82	6.75	1.50	8.82
Ferrum oxide	18.00	4.00	23.5	18.00	4.00	23.5	18.00	4.00	23.5	18.00	4.00	23.53
Stearic acid	1.17	0.26	1.5	1.17	0.26	1.5	1.17	0.26	1.5	1.17	0.26	1.53
Zincum oxide	0.98	0.20	1.3	0.98	0.22	1.3	0.98	0.22	1.3	0.98	0.22	1.28
Accelerators	2.11	0.47	2.7	2.11	0.47	2.7	2.11	0.47	2.7	2.11	0.47	2.7
Sulfur	1.29	0.29	1.7	1.29	0.29	1.7	1.29	0.29	1.7	1.29	0.29	1.69
Sage	3.44	0.85	5.00	6.89	1.70	10.00	13.77	3.40	20.00	20.66	5.10	30.00
**Samples with the Addition of Lucerne**												
	**5 PHR of Lucerne**			**10 PHR of Lucerne**			**20 PHR of Lucerne**			**30 PHR of Lucerne**		
**Component**	**M [g]**	**%**	**PHR**	**M [g]**	**%**	**PHR**	**M [g]**	**%**	**PHR**	**M [g]**	**%**	**PHR**
EPDM	31.90	7.09	41.70	31.90	7.09	41.70	31.90	7.09	41.70	31.90	7.09	41.70
EPDM off spec.	44.60	9.91	58.30	44.60	9.91	58.30	44.60	9.91	58.30	44.60	9.91	58.30
Paraffin oil	63.00	14.00	82.35	63.00	14.00	82.35	63.00	14.00	82.35	63.00	14.00	82.35
Calcium Carbonate	276.39	61.42	361.29	272.57	60.57	356.29	264.92	58.87	346.29	257.27	57.17	336.29
Poliethylene	6.75	1.50	8.82	6.75	1.50	8.82	6.75	1.50	8.82	6.75	1.50	8.82
Ferrum oxide	18.00	4.00	23.50	18.00	4.00	23.5	18.00	4.00	23.5	18.00	4.00	23.53
Stearic acid	1.17	0.26	1.50	1.17	0.26	1.50	1.17	0.26	1.50	1.17	0.26	1.53
Zincum oxide	0.98	0.22	1.30	0.98	0.22	1.30	0.98	0.22	1.30	0.98	0.22	1.28
Accelerators	2.11	0.47	2.7	2.11	0.47	2.7	2.11	0.47	2.7	2.11	0.47	2.7
Sulfur	1.29	0.29	1.70	1.29	0.29	1.70	1.29	0.29	1.70	1.29	0.29	1.69
Lucerne	3.44	0.85	5.00	6.89	1.70	10.00	13.77	3.40	20.00	20.66	5.10	30.00

**Table 3:** Results of the strength tests.

Samples with the Addition of Sage												
				0 PHR	5 PHR		10 PHR		20 PHR		30 PHR	
Property	Unit	Test Condition	Target	Value	Value	% Change	Value	% Change	Value	% Change	Value	% Change
Hardness	ShA	30 min, 170 °C	55–65	58.2	56.4	−3%	56.70	−3%	44.4	−24%	35.4	−39%
Spec. grav.	-	30 min, 170 °C	1.55–1.65	1.58	1.57	−1%	1.58	0%	1.34	−15%	1.20	−24%
ML	dNm	10 min, 175 °C	-	0.70	0.70	0%	0.71	1%	0.72	3%	0.72	3%
MH	dNm	10 min, 175 °C	-	7.86	7.61	−3%	7.10	−10%	5.16	−34%	4.69	−40%
MH-ML	dNm	-	>6.0	7.16	6.91	−3%	6.39	−11%	4.88	−32%	3.97	−45%
ts2	min	10 min, 175 °C	0.8–1.2	1.61	1.43	−11%	1.37	−15%	1.36	−16%	1.46	−9%
t90	min	10 min, 175 °C	-	7.27	6.53	−10%	5.75	−21%	4.34	−40%	3.72	−49%
MU	min	ML(1+4), 100 °C	40–50	43.06	44.55	3%	43.55	1%	44.11	2%	44.08	2%
Tsb	MPa	30 min, 170 °C	>4.0	4.65	4.13	−11%	3.85	−17%	2.03	−56%	1.51	−68%
Eb	%	30 min, 170 °C	>600	550	559	2%	574	4%	520	−5%	460	−16%
Color	-		<2.0	1.21	1.52	26%	1.15	−5%	2.35	94%	3.15	160%
**Samples with the Addition of Lucerne**												
				**0 PHR**	**5 PHR**		**10 PHR**		**20 PHR**		**30 PHR**	
**Property**	**Unit**	**Test Condition**	**Target**	**Value**	**Value**	**% Change**	**Value**	**% Change**	**Value**	**% Change**	**Value**	**% Change**
Hardness	ShA	30 min, 170 °C	55–65	58.2	56.4	−3%	53.70	−8%	35.6	−39%	41.3	−29%
Spec. grav.	-	30 min, 170 °C	1.55–1.65	1.58	1.59	0%	1.56	−1%	1.27	−20%	1.46	−8%
ML	dNm	10 min, 175 °C	-	0.70	0.70	0%	0.73	4%	0.75	7%	0.80	14%
MH	dNm	10 min, 175 °C	-	7.86	7.76	−1%	6.76	−14%	5.01	−36%	3.58	−54%
MH-ML	dNm	-	>6.0	7.16	7.06	−1%	6.03	−16%	4.26	−41%	2.78	−61%
ts2	min	10 min, 175 °C	0.8–1.2	1.61	1.36	−16%	1.28	−20%	1.31	−19%	1.71	6%
t90	min	10 min, 175 °C	-	7.27	6.33	−13%	5.28	−27%	3.70	−49%	2.81	−61%
MU	min	ML(1+4), 100 °C	40–50	43.06	44.30	3%	43.02	0%	43.79	2%	46.70	8%
Tsb	MPa	30 min, 170 °C	>4.0	4.65	3.82	−18%	3.57	−23%	1.53	−67%	1.33	−71%
Eb	%	30 min, 170 °C	>600	550	538	−2%	574	4%	382	−31%	470	−15%
Color	-		<2.0	1.21	0.94	−22%	1.58	31%	3.01	149%	3.37	179%

**Table 4:** The relationship between the tested parameters and the content of dried plant matter.

Dried Plant Content [PHR]	Sage				Lucerne			
	Hardness [ShA]	dM [dNm]	Tsb [MPa]	Eb [%]	Hardness [ShA]	dM [dNm]	Tsb [MPa]	Eb [%]
5	56.4	6.91	4.13	559	56.4	7.06	3.82	538
10	56.7	6.39	3.85	574	53.7	6.03	3.57	574
20	44.4	4.88	2.03	520	35.6	4.26	1.53	382
30	35.4	3.97	1.51	460	41.3	2.78	1.33	470

**Table 5:** Results after aging at 70° for 14 days.

Samples with the Addition of Sage													
				0 PHR		5 PHR		10 PHR		20 PHR		30 PHR	
Property	Unit	Test Condition	Target	Value	% Change	Value	% Change	Value	% Change	Value	% Change	Value	% Change
Hardness	ShA	30 min, 170 °C	55–65	65.2	12%	64.5	14%	65.40	15%	57.7	30%	46.1	30%
Tsb	MPa	30 min, 170 °C	>4.0	4.45	−4%	4.01	−3%	3.60	−6%	2.14	5%	2.24	48%
Eb	%	30 min, 170 °C	>600	447	−19%	452	−19%	439	−24%	420	−19%	460	0%
Color	-		<2.0	0.94	−22%	2.10	38%	2.23	94%	4.04	72%	6.80	116%
**Samples with the Addition of Lucerne**													
				**0 PHR**		**5 PHR**		**10 PHR**		**20 PHR**		**30 PHR**	
**Property**	**Unit**	**Test Condition**	**Target**	**Value**	**% Change**	**Value**	**% Change**	**Value**	**% Change**	**Value**	**% Change**	**Value**	**% Change**
Hardness	ShA	30 min, 170 °C	55–65	65.2	12%	66.8	18%	60.80	13%	38.20	7%	47.00	14%
Tsb	MPa	30 min, 170 °C	>4.0	4.45	−4%	4.39	15%	3.96	11%	1.60	5%	1.22	−8%
Eb	%	30 min, 170 °C	>600	447	−19%	483	−10%	512	−11%	301	−21%	132	−72%
Color	-		<2.0	0.94	−22%	1.67	78%	3.08	95%	5.62	87%	5.47	62%

**Table 6:** Socio-economic benefits of sage and lucerne usage in polymer-based composites.

Category	Description
Local Job Creation and Agricultural Development [34,35,36,37]	Utilizing natural fillers derived from plants can stimulate local agricultural sectors, especially in rural areas with suitable conditions for growing sage and lucerne. This change encourages small-scale farming and could create employment opportunities across multiple stages of the supply chain: Cultivation and Harvesting: Increased demand for plant fillers would boost crop production, creating seasonal and long-term jobs for farmers, laborers, and transport workers. Processing and Pre-Treatment: Post-harvest activities, such as drying, grinding, and refining plant materials for industrial use, could lead to the establishment of small processing facilities, driving local business growth. Distribution and Logistics: Expanding supply chains for natural fillers would generate jobs in logistics, warehousing, and material handling, benefiting regional economies.
Supply Chain Resilience and Resource Independence [34,35,36,37,38]	Relying on plant-based fillers reduces dependence on synthetic or fossil-derived materials, which are often subject to volatile market prices and geopolitical disruptions. By integrating locally grown and renewable materials, Producers mitigate raw material price fluctuations, stabilizing production costs and improving long-term financial planning. Regional economies gain autonomy, reducing reliance on imported fillers and strengthening domestic manufacturing. Circular economy models become viable—agricultural waste and non-food crops are repurposed as raw materials, promoting resource efficiency and reducing environmental strain.
Environmental and Societal Health Benefits [34,35,36,37,38]	The transition to bio-fillers contributes to public health and environmental sustainability: Reduced Pollution: Natural fillers generate less pollution during production than synthetic alternatives, lowering emissions and minimizing industrial waste. Safer Work Environments: Using plant-based materials can decrease workers' exposure to hazardous chemicals and fine particulate matter, reducing occupational health risks. Consumer Health and Perception: End consumers may view bio-composite products as healthier and safer, especially in applications like footwear, household goods, or children’s toys, driving market demand for eco-friendly alternatives.
Market Competitiveness and Innovation Opportunities [34,39,40,41,42]	With growing consumer awareness of sustainability and the pressure of environmental regulations, companies that pioneer plant-based composites gain a competitive advantage. Using fillers like sage and lucerne not only enhances a company's green image, but can also Open new market segments: Eco-conscious consumers are willing to pay a premium for sustainable products, expanding business opportunities. Attract funding and partnerships: Innovation in bio-composites aligns with government and EU funding programs supporting circular economy initiatives. Drive technological innovation: The search for optimal filler compositions (e.g., 5 or 10 PHR) could lead to breakthroughs in materials science, with applications beyond rubber composites, such as in coatings, packaging, or biomedical materials.
Long-Term Cost Efficiency and Lifecycle Savings [34,43,44,45,46]	Although bio-fillers may slightly reduce mechanical properties, they offer long-term cost advantages: Lower Disposal Costs: Natural fillers are biodegradable or easier to process at the product’s end of life, reducing landfill fees and simplifying waste management. Regulatory Compliance: As carbon taxes and environmental restrictions tighten, bio-composite producers could avoid penalties and benefit from incentives for low-impact manufacturing. Material Circularity: Residual plant waste from production could be repurposed as biomass energy or compost, creating closed-loop systems that reduce costs and resource consumption.

## Data Availability

The original contributions presented in this study are included in the article. Further inquiries can be directed to the corresponding authors.
